# Tumor-Induced Local and Systemic Impact on Blood Vessel Function

**DOI:** 10.1155/2015/418290

**Published:** 2015-12-07

**Authors:** J. Cedervall, A. Dimberg, A-K. Olsson

**Affiliations:** ^1^Department of Medical Biochemistry and Microbiology, Science for Life Laboratory, Biomedical Center, Uppsala University, P.O. Box 582, 75123 Uppsala, Sweden; ^2^Department of Immunology, Genetics and Pathology, Science for Life Laboratory, Rudbeck Laboratory, Uppsala University, 75185 Uppsala, Sweden

## Abstract

Endothelial dysfunction plays a role in several processes that contribute to cancer-associated mortality. The vessel wall serves as a barrier for metastatic tumor cells, and the integrity and activation status of the endothelium serves as an important defense mechanism against metastasis. In addition, leukocytes, such as cytotoxic T-cells, have to travel across the vessel wall to enter the tumor tissue where they contribute to killing of cancer cells. Tumor cells can alter the characteristics of the endothelium by recruitment of leukocytes such as neutrophils and macrophages, which further stimulate inflammation and promote tumorigenesis. Recent findings also suggest that leukocyte-mediated effects on vascular function are not limited to the primary tumor or tissues that represent metastatic sites. Peripheral organs, such as kidney and heart, also display impaired vascular function in tumor-bearing individuals, potentially contributing to organ failure. Here, we discuss how vascular function is altered in malignant tissue and distant organs in individuals with cancer and how leukocytes function as potent mediators of these tumor-induced effects.

## 1. Introduction

**Figure 1:**
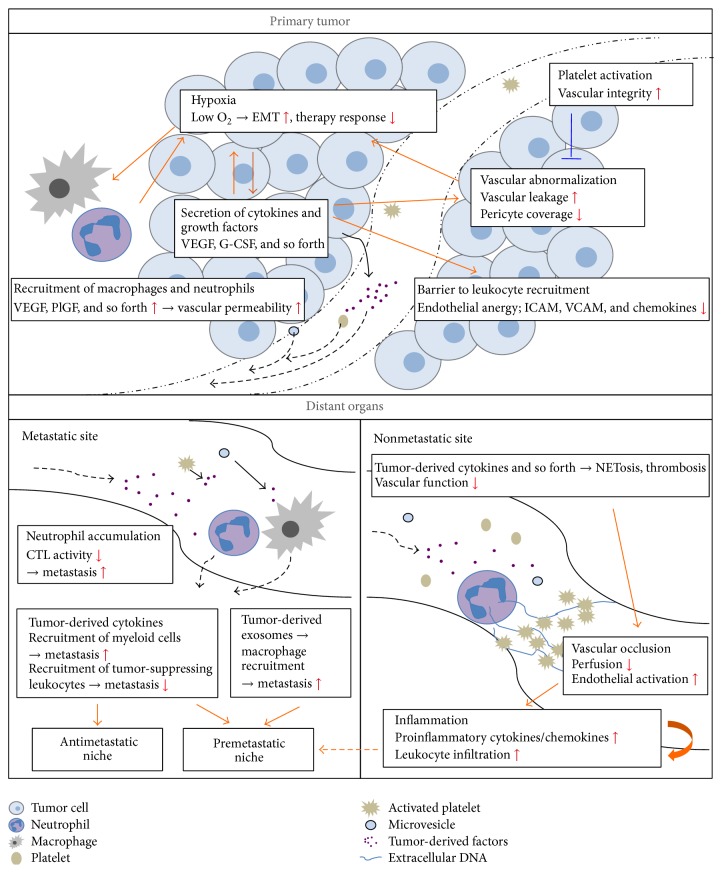
Altered function of blood vessels in tumor tissue and distant organs in individuals with cancer. Vascular function is impaired both at local tumor level and systemic level in an individual with cancer. The primary tumor secretes proangiogenic growth factors that contribute to vascular abnormalization with enhanced permeability and anergic endothelial cells within the tumor. The poor vascular function leads to hypoxia and subsequent recruitment of macrophages and neutrophils that further contribute to vascular permeability by secretion of additional proangiogenic factors. Hypoxia stimulates tumor invasiveness by induction of EMT and contributes to impaired therapy response. Effects on the vasculature are not limited to the actual tumor, but altered vascular function is also found in distant organs of tumor-bearing individuals. Tumor cell-derived cytokines are spread throughout the body in plasma or as cargo in platelets or microvesicles and can contribute to formation of pre- or antimetastatic niches in organs that exert sites for metastasis. These effects are mainly mediated by recruitment of leukocytes to the metastatic sites, which prepare the microenvironment to facilitate metastatic colonization. Furthermore, tumor-derived factors stimulate NETosis and thrombosis in distant organs leading to vascular occlusion and systemic inflammation also in organs that are not sites for metastasis.

During the last decades, it has become increasingly clear that cancer is a complex disease with systemic effects, which contribute significantly to the mortality. Indeed, the absolute majority of cancer-related deaths is caused by tumor-induced systemic events, such as metastasis and thrombosis. The vasculature is central in these processes, since it is a transport system that spans all organs of the individual. Via this route, tumor-derived factors, as well as disseminating tumor cells, can spread to distant organs, where they contribute to the disease state directly by promoting formation of metastases or indirectly, for example, by induction of thrombosis. In this review, we discuss how endothelial function is affected in individuals with cancer and how the primary tumor dictates these alterations by activation and recruitment of leukocytes. Furthermore, the consequences for tumor progression as well as distant organ function and systemic inflammation in the afflicted individual will be addressed. A summary of the effects discussed in the text can be found in Figure 1.

Tumors stimulate and recruit leukocytes not only to the local tumor microenvironment, but also to other sites in an individual with cancer. For example, tumors express cytokines and growth factors, such as G-CSF and VEGF, which modulate leukocyte stimulation and trafficking over the endothelium. The effects of these tumor-produced factors are however not limited to the site of the primary tumor. Tumor-derived cytokines and growth factors can spread systemically by free transport in the blood or be distributed by carriers such as platelets or microvesicles [1, 2]. Several of these tumor-derived factors affect the integrity and function of the endothelium, either directly or secondary to changes in endothelial-leukocyte interactions.

## 2. Local Effects in the Tumor Microenvironment

Compared to healthy vessels under physiological conditions, the tumor vasculature is frequently poorly functional with permeable and leaky vessels, and the hierarchical organization is often lost and replaced by a chaotic vascular system with disturbed blood flow [3]. This typical characteristic of the tumor vasculature has extensive impact on tumor progression. Poor vascular function leads to intermittent or chronic hypoxia, which affects the tumor phenotype directly and contributes to increased tumor invasiveness and metastasis by induction of Epithelial-Mesenchymal Transition (EMT) [4]. The vascular function also affects the response to therapy, since good vascular perfusion is crucial for delivery of therapeutic substances to the tumor, and maintained oxygen tension and physiological pH are required for efficient killing of tumor cells by radiation and chemotherapy. Importantly, the vasculature regulates recruitment of leukocytes to the tumor, and the recruited leukocytes in turn affect vascular function.

### 2.1. The Tumor Endothelial Barrier

During inflammation and wound healing, proinflammatory cytokines stimulate endothelial cells to upregulate adhesion molecules and chemokines that together mediate the capture and extravasation of leukocytes from the blood to the tissue. Tumor endothelial cells are anergic in the sense that they respond poorly to proinflammatory stimulation. This is at least in part due to constant stimulation by proangiogenic factors, including FGF and VEGF, which inhibit TNF-*α*-induced upregulation of ICAM, VCAM, and chemokines through interference with NF-kappaB-signaling pathways [5–9]. Consequently, antiangiogenic therapy can restore adhesion molecule expression in tumor endothelial cells and induce leukocyte recruitment [8, 9]. The tumor vessels may also block the activation of T-cells that are recruited to the tumor tissue by expressing inhibitory molecules such as PDL1 and IDO1 or directly induce T-cell apoptosis by expression of death-receptor family members including TRAIL or FASL [10, 11]. Thus, tumor endothelial gene expression may significantly affect the quantity and activation of leukocytes recruited to the tissue. Indeed, endothelial expression of the Endothelin B receptor has been shown to inhibit T-cell recruitment in ovarian cancer and decrease efficacy of cancer immunotherapy [12]. The location and quantity of tumor-promoting macrophages and tumor-inhibiting cytotoxic T-cells are predictive of survival in many types of solid tumors [13], and the success of cancer immunotherapy strictly depends on efficient recruitment of tumor-targeting immune cells [14]. Therefore, the endothelial barrier represents an attractive target for treatment of cancer [15]. Importantly, the recruited immune cells also affect tumor vessel quality and gene expression, as delineated below.

### 2.2. Tumor-Promoting Effects

Cells of the innate immune system, such as macrophages and neutrophils, are crucial regulators of angiogenesis and vascular properties in the tumor microenvironment. Macrophages are often classified into two subpopulations: the proinflammatory M1 macrophages with tumor-suppressing properties and the immunosuppressive M2 macrophages considered as tumor promoters. However, it is now emerging that the division into two distinct macrophage subpopulations is too simplified and that macrophages likely display a spectrum of phenotypic variation [16]. Macrophage recruitment is stimulated by hypoxia and infiltration into hypoxic tumor areas is guided by tumor-derived factors such as VEGF or CCL2 [17–19]. Upon arrival, the hypoxic tumor microenvironment stimulates macrophages to produce VEGF and MMPs, which promotes angiogenesis and contributes to permeability of the tumor vasculature. In addition, macrophages produce numerous other growth factors (PlGF, FGF, PDGF, M-CSF, and TGF-*β*) and cytokines (IL-1, IL-8, and TNF-*α*) that stimulates angiogenesis and activates the endothelium [20, 21].

Similar to macrophages, neutrophils are potent regulators of tumor angiogenesis. Recruitment and transendothelial migration of neutrophils are mediated via chemokine signaling, and tumor-derived CXCL8 has been suggested to play an important role in these processes [22, 23]. At the tumor site, TNF-*α* can induce direct release of VEGF from the neutrophils [24]. Furthermore, neutrophils secrete MMP-9, which contribute to increased release of VEGF bound to the extracellular matrix and further promote angiogenesis and vessel permeability [25, 26]. Innate immune cells such as macrophages and neutrophils hence contribute significantly to the permeable and leaky vascular phenotype observed in tumors, mainly by increasing the concentration of bioavailable VEGF in the microenvironment.

Another cell type that has been shown to maintain the endothelial barrier and increase tumor growth is the platelet. In tumor vessels, platelets play an important role in protecting tumor vessels from hemorrhage [27–29]. Depleting mice with established tumors from platelets results in bleeding specifically in the tumor tissues [27]. Furthermore, it has been demonstrated that inflammation and associated leukocyte infiltration are causing the tumor hemorrhage during thrombocytopenia [27, 30]. If neutrophil infiltration into the tumor tissue is reduced by genetic deletion of beta2-integrin (CD18−/−), tumor hemorrhage is suppressed after platelet depletion [28, 30]. A role for macrophages in tumor-induced bleeding during thrombocytopenia was also described [28]. Recently, the importance of leukocytes was further supported by a study showing that diapedesis of neutrophils through the endothelium is crucial for hemorrhage during thrombocytopenia in several mouse models of inflammatory disease [31]. It was further demonstrated that the vessel-protective effect of platelets is mediated by secretion of platelet granules rather than platelet adhesion to the endothelium [27]. Platelet granule secretion was suggested to provide factors that suppress permeability, such as serotonin and angiopoetin-1, and hence balance the permeability promoting effect of VEGF. It has also been demonstrated that platelets contribute to integrity and function of the tumor vasculature by affecting pericyte coverage [32]. Platelet depletion of transgenic RIP1-Tag2 mice with insulinoma resulted in significantly decreased pericyte coverage of the tumor vasculature and severely impaired perfusion. How platelets support pericyte coverage of the vasculature in a tumor remains to be explored.

### 2.3. Tumor-Suppressing Effects

Infiltration of innate immune cells may not only play a tumor-promoting role but can also under certain conditions and in some types of cancers exert tumor-suppressing effects. While a high number of tumor infiltrating neutrophils correlate with poor survival in a variety of different tumors types [33–37], the opposite has been demonstrated, for example, in patients with gastric cancer [38]. Furthermore, tumor suppressive effects of infiltrating neutrophils have also been demonstrated in various experimental models of breast cancer. Using an* in vitro* approach, it was shown that neutrophil-derived elastase (NE) was taken up by breast cancer cells and contributed to T lymphocyte-mediated tumor cell lysis [39]. In an orthotopic mouse model of breast cancer, neutrophils were further found to suppress metastasis by preventing metastatic seeding in the lungs [40]. In addition to the more prominent proangiogenic role of neutrophils described earlier, they also contain antiangiogenic mediators such as NE that can suppress VEGF-mediated angiogenesis and leakage and hence support integrity of the tumor vasculature [41–43]. The high number of tumor infiltrating macrophages correlates in the majority of tumor types with poor prognosis, reflecting the fact that macrophages mainly exert tumor-promoting effects. Some reports however suggest a correlation between high level of macrophage infiltration and positive prognosis in patients with osteosarcoma and gastric cancer [44, 45]. The tumor-suppressing effects are mediated by proinflammatory macrophages, often referred to as M1 macrophages. Macrophages of the proinflammatory phenotype, induced, for example, by IFN-*γ*, produce Reactive Oxygen Species (ROS) and proinflammatory cytokines such as IL-1*β* and IL-6 that contributes to activation of the endothelium [46]. This further promotes recruitment of cytotoxic T-lymphocytes to the tumor microenvironment, which can suppress growth of the tumor.

The adaptive immune system has mainly been attributed a tumor-suppressive role. However, B-lymphocytes may support inflammation-associated epithelial carcinogenesis [47] and regulatory T-lymphocytes are frequently induced in the tumor microenvironment and suppress the antitumorigenic activity of cytotoxic T-lymphocytes [48]. Classifying tumors according to the “immunoscore,” which takes into account the location and prevalence of different leukocyte subsets in the tumor microenvironment, can be used to predict patient survival for several solid tumor types [49]. Immune checkpoint therapy, involving reactivation of cytotoxic T-cells with antibodies targeting CTLA4 or PDL1/PD1, has recently gained success in the clinical treatment of cancer [50].

## 3. Systemic Effects on Peripheral Vasculature and Organ Function in Individuals with Cancer

Leukocyte-mediated effects on vascular function are not limited to the local tumor microenvironment but appear to reach far beyond the actual tumor. Altered endothelial integrity and recruited immune cells can affect malignant progression directly by altering the milieu in organs that represent sites for metastasis—even before the tumor cells arrive. Furthermore, recent data show that vascular function is impaired in distant organs not directly affected by either the primary tumor or metastases in mice with cancer.

### 3.1. Tumor-Induced Effects on Organs that Represent Metastatic Sites

Metastasis, responsible for the absolute majority of cancer-related deaths, is a complex and challenging process for the tumor cells. Indeed, only a small fraction of the disseminating tumor cells will eventually succeed in establishing a secondary tumor in a distant organ.

It has however been demonstrated that the primary tumor can facilitate metastatic colonization by orchestrating systemic processes that prepare the distant organ before the metastatic tumor cells arrive, that is, creating a premetastatic niche. This was first suggested more than a decade ago, when several studies showed that tumor-derived VEGF-A, PlGF, TGF-*β*, and TNF-*α* contribute to recruitment of CD11b+ myeloid cells to the lungs in tumor-bearing mice before tumor dissemination and that this results in enhanced recruitment of metastatic cells to the lung [51–53]. Since then, additional tumor-derived factors (LOX, CCL2, and VCAN) have been shown to stimulate recruitment of bone-marrow-derived cells (BMDCs) and hence contribute to formation of the premetastatic niche in a similar manner [54–56]. Besides a few exceptions [56–58], these studies focus on lung tissue, and whether the described effects occur also in other organs with metastatic growth, or even throughout the body, has not been firmly established. Some lines of evidence do support that this is a general phenomenon. A few years ago, a study revealed that systemic inflammation, induced by arthritis, enhanced metastasis in a transgenic mouse model of mammary carcinoma [59]. This effect was observed not only in lung but also in bone marrow, indicating that systemic inflammation may be a general promoter of metastasis. This hypothesis was recently confirmed by data from Coffelt and colleagues demonstrating that systemic neutrophil expansion and accumulation in multiple organs occurs in a mouse mammary tumor model with spontaneous lung metastases [60]. These tumor-induced neutrophils suppressed the ability of CD8+ cytotoxic T-cells to kill tumor cells, thus resulting in an increased metastatic burden. Another recent paper also reports on systemic accumulation of neutrophils in peripheral organs in mice with distinct tumor types such as mammary carcinoma and insulinoma [61]. Furthermore, upregulation of leukocyte adhesion markers as well as proinflammatory cytokines such as IL-1*β*, IL-6, and CXCL1 was detected in the kidney tissue, indeed supporting an ongoing systemic inflammation in individuals with cancer [61].

While the factors responsible for formation of the premetastatic niche may be distributed freely in the circulation, they were recently also reported to spread as cargo in tumor-derived exosomes. This mechanism was first described in mouse models of melanoma [62, 63] but was recently demonstrated also in mice with pancreatic ductal adenocarcinoma (PDAC) [64]. Costa-Silva and colleagues showed that primary tumor-derived exosomes promote enhanced metastatic burden in the liver. This effect was mediated by increased macrophage recruitment from the bone marrow, induced by macrophage migration inhibitory factor (MIF) expressed in the exosomes [64].

In contrast to the situations discussed above, some reports suggest that tumor-derived factors can stimulate leukocytes to function as metastatic suppressors and as such contribute to formation of antimetastatic niches. It was, for example, demonstrated a few years ago that tumor-entrained neutrophils (TENs), upon stimulation by tumor-derived G-CSF and CCL2, prevent lung metastasis [40].

### 3.2. Tumor-Induced Effects on Organs that Do not Represent Sites for Metastases

While a vast amount of research has focused on organs that represent sites for metastasis, less is known about cancer-induced effects in distant organs that are not affected by either primary or secondary tumor growth. However, one recently published paper demonstrate that mice with cancer display significantly impaired function of the vasculature in heart and kidney, organs that are not targets for metastasis in the tumor models used [61]. Furthermore, it was shown that the reduced peripheral vascular function was caused by formation of Neutrophil Extracellular Traps (NETs), which occlude peripheral vessels in tumor-bearing mice [61]. NET formation (NETosis) was first described in 2004 as a novel mechanism used by neutrophils to fight bacterial infections [65]. During NETosis, neutrophils secrete their chromatin together with proteases such as Myeloperoxidase (MPO) and Neutrophil Elastase (NE). However, NETs are also highly prothrombotic, mainly due to the negatively charged chromatin and associated histones. In this way, neutrophils undergoing NETosis may also stimulate thrombosis, leading to further vascular occlusions [66, 67]. Removal of the intravascular NETs by DNase treatment restored functionality of the peripheral vessels in tumor-bearing mice [61]. In addition to occluding the vessels, NETs may damage the vasculature in other ways. It was previously shown that NETs have cytotoxic effects on the endothelium and that they directly induce endothelial damage in other pathological conditions [68–70].

Organ failure in general, and acute renal failure (ARF) in particular, is a cause of substantial morbidity in cancer patients and is characterized by hypoperfusion of the kidney vasculature [71]. The mechanisms behind tumor-induced organ failure are however poorly studied. Systemic intravascular NET formation offers a potential explanation for how these fatal effects occur.

A link between cancer and NETosis was first demonstrated in 2012, when Demers and colleagues showed that cancer is a predisposing factor for NETosis in mice and that this subsequently contributes to thrombosis [72]. Formation of NETs can also directly contribute to malignant progression. In mice with liver tumors exposed to sepsis, NETs that formed due to the infection were reported to sequester circulating tumor cells and promote metastasis [73]. These data imply that an infection is a potential risk factor for metastasis. It is also possible that NETs facilitate metastasis by inducing inflammation and upregulation of adhesion molecules in peripheral vessels [61], thereby offering a route for extravasation in a secondary organ. It has, for example, been shown that VCAM-1 can be used by tumor cells to adhere to the endothelium and hence facilitates transendothelial migration [74]. Furthermore, tumor cell expression of E-selectin binding ligands such as Sialyl Lewis (a) has been correlated to malignancy and prognosis in the clinic [75, 76]. Whether NETs really promote extravasation remains to be explored.

## 4. Conclusion and Perspective

Endothelial activation and vascular integrity are crucial regulators of tumor progression and related systemic effects (see summary in Figure 1). Serving as a barrier for infiltrating leukocytes and metastasizing tumor cells, the endothelium plays an important role in protecting us from the fatal processes responsible for cancer-related deaths. When designing new cancer therapies, it is therefore of utmost importance to consider potential effects on the vasculature in the local tumor microenvironment, as well as in peripheral organs. For immunotherapeutic approaches it would be beneficial to enhance endothelial transmigration of cytotoxic T-lymphocytes into the tumor, to improve the killing of tumor cells. On the systemic level, inflammation and endothelial activation should probably be kept as low as possible, to avoid tumor extravasation into secondary tissues.
